# An Account of Diseases in an Eastern District of London from July 20, to August 20, 1809

**Published:** 1809-09

**Authors:** 


*An Account of Diseases in an Eastern District of London from July 20, to August 20, 1809.*

ACUTE DISEASES.

| | |
|---|---|
| Febris Intermittens - - | 1 |
| Cynanche Tonsillaris - | 4 |
| Catarrhus - - - - - | 3 |
| Dysenteria - - - - | 2 |
| Rheumatismus Acutus | 2 |

CHRONIC DISEASES.

| | |
|---|---|
| Tussis - - - - - - - | 5 |
| Tussis cum Dyspnœa - | 4 |
| Hæmoptysis - - - - | 2 |
| Pleurodyne - - - - | 3 |
| Hepatitis Chronica - - | 2 |
| Hydrothorax - - - - | 4 |
| Icterus - - - - - - | 1 |
| Leucorrhœa - - - - | 4 |
| Dysuria Ardens - - - | 3 |
| Menorrhagia - - - | 4 |
| Amenorrhœa - - - - | 3 |
| Dysmenorrhœa - - - | 1 |
| Enterodynia - - - - | 4 |
| Hypochondriasis - - - | 3 |
| Rheumatismus Chronicus | 9 |

PUERPERAL DISEASES.

| | |
|---|---|
| Menorrhagia Lochialis | 3 |
| Abscessus Mammæ - - | 4 |
| Rhagas Papillæ - - - | 5 |
| Dolor Post Partum - - | 7 |

INFANTILE DISEASES.

| | |
|---|---|
| Convulsio - - - - - | 2 |
| Febris Mesenterica - - | 3 |
| Diarrhœa - - - - - - | 7 |
| Dentitio - - - - - - | 4 |
| Vermes - - - - - - - | 3 |

In several former Reports, a great number of cases of pneumonic inflammation were taken notice of, several of which, it was observed, would probably terminate in hydrothorax and anasarca. This has been verified in a number of instances, some of which still continue under medical treatment. In these cases a variety of circumstances occur. In some of them the original disease so far disappears, that the patient returns to his former habits, and any inconvenience that he feels is attributed to weakness induced by the disease, or by the different evacuations necessary in the course of cure. After some time he finds that his feet and ancles occasionally swell, particularly towards evening, and this appearance gradually extends up his legs and thighs, and in the morning his face and upper extremities appear enlarged. After some time difficulty of breathing occurs, and the disease assumes the character of hydrops pectoris, which too frequently proceeds to a fatal termination. In one of the cases referred to in the list, considerable relief was experienced from vesications forming in the lower extremities, from which a large quantity of water has been discharged, and the anasarcous swelling, which was becoming very general, considerably diminished. In another case, by the advice of a female friend, blisters were applied to the legs, which produced inflammation and mortification that soon put a period to life.

